# Study on the improvement of grouting stone properties in coal mine goafs using combined denitrifying bacteria

**DOI:** 10.1098/rsos.240993

**Published:** 2024-09-25

**Authors:** Hao He, Nengxiong Xu, Yan Qin, Tianchi Xu, Yuxi Guo

**Affiliations:** ^1^ School of Engineering and Technology, China University of Geosciences (Beijing), Xueyuan Road 29, Beijing 100083, People’s Republic of China; ^2^ Engineering and Technology Innovation Center for Risk Prevention and Control of Major Project Geosafety, MNR, Beijing, People’s Republic of China

**Keywords:** microbially induced calcite precipitation, coal mine goaf, grouted stone body, physical and mechanical properties

## Abstract

Grouting can effectively reduce residual deformation of coal mine goafs, but fly ash grouting materials suffer from poor flowability and slow early strength development. Microbially -induced calcite precipitation (MICP), with its high environmental compatibility and minimal disturbance to geotechnical bodies, effectively improves the injectability of grouting slurry in goafs. This study combined *Castellaniella denitrificans* and *Sporosarcina pasteurii* to induce calcite precipitation, preparing cement–fly ash slurry with varying water–solid ratios, solid ratios and denitrifying bacteria concentrations. The physical properties of the slurry and the mechanical properties of the grouted stone bodies under sealed curing conditions were measured. Results show that the dual-bacteria MICP improves stone body performance by enhancing cohesive, frictional and interlocking forces, so that the strength of the stone bodies cured by MICP increased rapidly within 7 days, and the strength reached the standard 2.03 MPa at 28 days under conditions of low solid ratio and high water–solid ratio, with the best compressive strength at a denitrifying bacteria concentration with an optical density of 0.8 at 600 nm wavelength. At a water–solid ratio of 1 : 1.2 and a solid ratio of 15%, initial and final setting times were 67.2 and 96 h, respectively, which prolonged the initial setting time and final setting time by nearly 70% and 110% compared with that of the slurry without MICP treatment, indicating that MICP enhances slurry fluidity, providing more time for grouting construction in goafs.

## 1. Introduction

Extensive mining and utilization of coal mines in China have resulted in the formation of large areas of goafs underground in coal mining regions [1]. The roof structures of these goafs are unstable, which easily triggers geological hazards such as ground fissures, surface subsidence and landslides [2]. Pressure grouting is one of the commonly used methods for goaf treatment [3]. This method first drills holes into the goaf and then uses artificial pressurization to inject a mixture of fly ash, clay, cement and sand, proportionally mixed, into the fractures of the fragmented rock mass in the goaf. This mixture fills the voids and cements the remaining coal pillars and fragmented rock mass, thereby reducing voids and deformation [4].

Currently, commonly used grouting materials face issues such as insufficient stability, low strength reliability and significant environmental pollution [5]. For instance, single-component cement grouting materials have poor stability and are prone to segregation [6]; slag may contain heavy metals and other harmful substances [7]. However, fly ash, as a grouting and filling material for coal mine goafs, has advantages such as a wide range of sources, low cost and stable physico-chemical properties. Its filling effect in coal mine filling projects has been widely demonstrated and applied [8,9]. Nonetheless, fly ash-based grouting materials encounter problems like poor flowability and slow early strength development during use. Therefore, there is an urgent need to find a green, environmentally friendly grouting slurry with good flowability and effective filling and reinforcement properties.

Microbially induced calcite precipitation (MICP) technology utilizes the metabolic products of microorganisms to engage in a series of biochemical reactions with the surrounding environment, resulting in the formation of calcium carbonate precipitates. This process improves the mechanical and hydraulic properties of the soil (such as permeability, collapsibility and erosion resistance) [10,11]. MICP technology has been widely applied in various fields, including soil reinforcement, concrete repair, historical building preservation and heavy metal pollution remediation. Van Paassen *et al*. [12] conducted large-scale sand foundation reinforcement experiments, achieving an average calcium carbonate content of 110 kg m^−3^ in the sand foundation after 16 consecutive days of grouting, and the uniaxial compressive strength (UCS) of the cemented sand reached 0.7−12.4 MPa, significantly improving the bearing capacity and stiffness of the sand foundation. Cheng *et al*. [13] applied MICP for sand foundation reinforcement and found that the liquefaction resistance of the soil improved by MICP grouting was superior to that of traditional reinforcement methods during moderate to strong earthquakes. Hassanin *et al*. [14] combined *Bacillus subtilis*, fly ash and polyvinyl alcohol (PVA) fibres to achieve autogenous and autonomous self-healing techniques in concrete, and the coupled action of the bacteria with the PVA fibres showed excellent performance in maintaining the long-term durability of the repaired concrete. Jongvivatsakul *et al*. [15] applied MICP technology to the repair of cracked concrete, increasing its compressive strength by 43% and enhancing the watertightness of the concrete. Rodriguez-Navarro *et al*. [16] applied MICP for the protection and consolidation of porous carbonate rocks in sculptures and architectural heritage, utilizing *Myxococcus xanthus*-induced calcium carbonate precipitation to effectively prevent weathering and erosion of the stone. Kang *et al*. [17] studied the application of MICP in heavy metal pollution remediation, finding that calcium carbonate precipitation fixed heavy metal ions, achieving a remediation rate of 98.3% for lead and 85.4% for cadmium within 48 h, significantly reducing the concentration of heavy metals in soil and water. Most studies on MICP technology, both domestically and internationally, use aerobic bacteria. However, underground goafs are often located tens to hundreds of metres below ground, mostly in anoxic environments [18]. Common aerobic urease bacteria, such as *Sporosarcina pasteurii*, experience inhibited MICP processes under anoxic conditions, with observed trace precipitation primarily due to urease produced during aerobic cultivation [19]. Thus, the urease hydrolysis-based MICP reaction is not suitable for direct application in underground goafs. There is relatively little research on the use of MICP technology in goafs, making the exploration of microorganisms and their metabolic pathways better suited to anoxic environments an important research direction.

Denitrification (equation 1.1) is one type of MICP process. Under anoxic conditions, bacteria use organic acid ions, such as acetate, as electron donors to reduce nitrate ions to harmless nitrogen gas while generating calcium carbonate crystals. Compared with other MICP reaction mechanisms (iron and sulphate reduction) this reaction has the unique advantages of being green, non-polluting, low cost and easy to cultivate. Li *et al*. used a mixture of *Castellaniella* denitrificans and *S. pasteurii* for sealed tailings cementation, showing that the UCS and shear strength of the samples increased by 29.9% and 24.4%, respectively, compared with using *S. pasteurii* alone [20]. Facultative anaerobic bacteria can overcome the low activity problem of aerobic bacteria in anoxic underground environments [21], while the urease hydrolysis function of aerobic bacteria can partially compensate for potential shortcomings of denitrifying MICP, such as lower calcium carbonate production rate, less precipitation and uneven distribution [20,22].


(1.1) $$CH_{3}COO^{-}\;\left(aq\right)+1.6NO_{3}^{-}\;\left(aq\right)+2.6^{+}\;\left(aq\right)\overset{Denitrifying\;bacteria}{\to }\;2CO_{2}(g)+0.8N_{2}(g)+2.8H_{2}O.$$

This study combines *C. denitrificans* and *S. pasteurii* to induce calcium carbonate precipitation, conducting a three-factor, three-level orthogonal experiment. Different water–solid ratios, solid ratios and concentrations of denitrifying bacteria (*C. denitrificans*) were used to prepare cement–fly ash grouting slurry. Six physical properties—density, plastic viscosity, initial setting time, final setting time, precipitation rate and stone rate—were measured, along with the early and late UCS of the grouted stone bodies under sealed curing conditions. The study indicates that under anoxic conditions, the combination of the two bacteria can effectively cement the grouted stone bodies. The stone body strength meets the requirements of the ‘JTG/T D31-03-2011 Technical Specifications for Highway Design and Construction in Goafs’^1^ while also increasing the fluidity of the slurry and reducing its setting time. This has significant reference value for future similar engineering practices in coal mine goafs.

## 2. Materials and sample preparation

### 2.1. Materials

#### 2.1.1. Microbes

The denitrifying bacterium *C. denitrificans* selected in this study is a Gram-negative facultative anaerobe, and the bacterium *S. pasteurii* is a Gram-positive aerobic bacterium. Their strain numbers are CGMCC1.10720 and CGMCC1.3687, respectively, both of which were purchased from the China General Microbial Strain Collection and Management Centre (CGMCC). The culture medium formulations for these strains are shown in table 1.

**Table 1.** Medium formulations.

microbe	*C. denitrificans*				*S. pasteurii*				
reagent formulation	peptone	beef extract	NaCl	pH	casein peptone	soy peptone	NaCl	urea	pH
	10 g l^−1^	3 g l^−1^	5 g l^−1^	7.0	15 g l^−1^	5 g l^−1^	5 g l^−1^	20 g l^−1^	7.3

**Figure 1.**
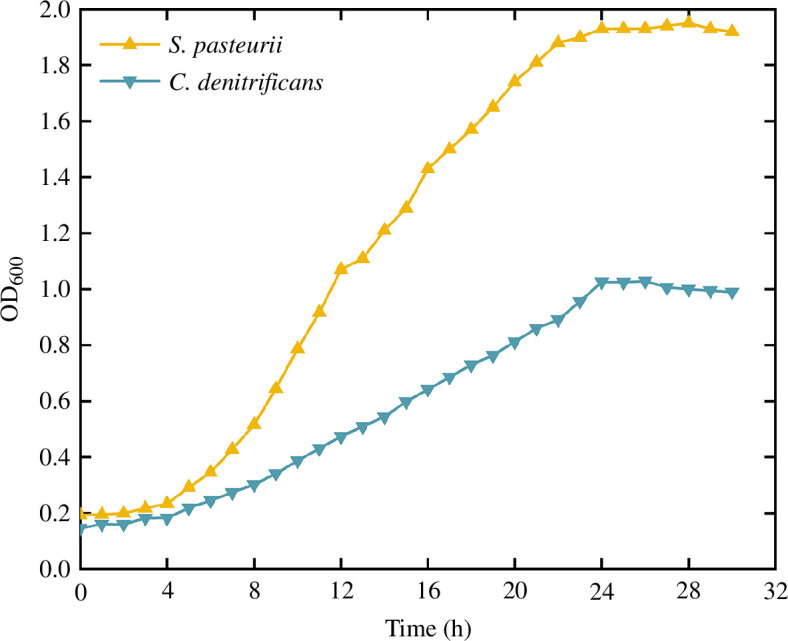
Microbial growth curve.

The prepared culture medium was sterilized by high-pressure steam at 120°C and 1 MPa for 30 min. After cooling, the activated bacteria were inoculated into the corresponding culture medium at a mass ratio of 1 : 100 in a clean bench. The inoculated cultures were then oscillated at 30°C and 140 r.p.m. The bacterial concentration was characterized by the absorbance of the bacterial liquid at a wavelength of 600 nm (OD_600_) [20,23]. During bacterial cultivation, the concentrations of *C. denitrificans* and *S. pasteurii* were measured every hour. As shown in figure 1, the growth curve of *C. denitrificans* and *S. pasteurii* within 30 h indicated that the concentrations of both bacteria reached their maximum values at 24 h, which were 1.02 and 1.93, respectively. Therefore, the bacterial oscillation cultivation time in this study was set to 24 h.

#### 2.1.2. Cementation solution

According to the reactions of urease hydrolysis and denitrification, the cementation solution was prepared using calcium nitrate, calcium acetate, calcium chloride, urea and deionized water. The concentration ratio of calcium acetate to calcium nitrate was maintained at 1 : 1.6 to ensure consistency with the denitrification MICP reaction. Regarding the selection of calcium ion concentration in the cementing solution, it was considered that the optimal calcium ion concentration for *C. denitrificans* is generally 1.5 mol l^−1^ [24], while for *S. pasteurii*, the best cementing effect is usually achieved with a calcium ion concentration of 0.6 mol l^−1^ [25]. Therefore, the total calcium ion concentration was set to 2.1 mol l^−1^. The specific proportions are shown in table 2. The components were dissolved in deionized water without the need for additional nutrients.

**Table 2.** Proportion of cementation solution.

chemical reagent	Ca(NO_3_)_2_	Ca(CH_3_COO)_2_·H_2_O	CaCl_2_	urea
concentration (mol l^−1^)	0.6	0.375	1.125	0.6

#### 2.1.3. Fly ash and cement

The grade I fly ash and 425# ordinary Portland cement used in this study were purchased from Baorun Refractory Material Co., Ltd, Gongyi City, China, and Shandong Fujian Building Materials Co., Ltd, respectively. Their chemical compositions are shown in table 3.

**Table 3.** Main chemical components of fly ash and cement (%).

materials	CaO	SiO_2_	Al_2_O_3_	Fe_2_O_3_	MgO	Na_2_O	SO_3_
fly ash	5.35	48.1	30.2	3.85	2.05	0.55	0
cement	51.42	24.99	8.26	4.03	3.71	0.2	2.51

### 2.2. Sample preparation method

**Figure 2.**
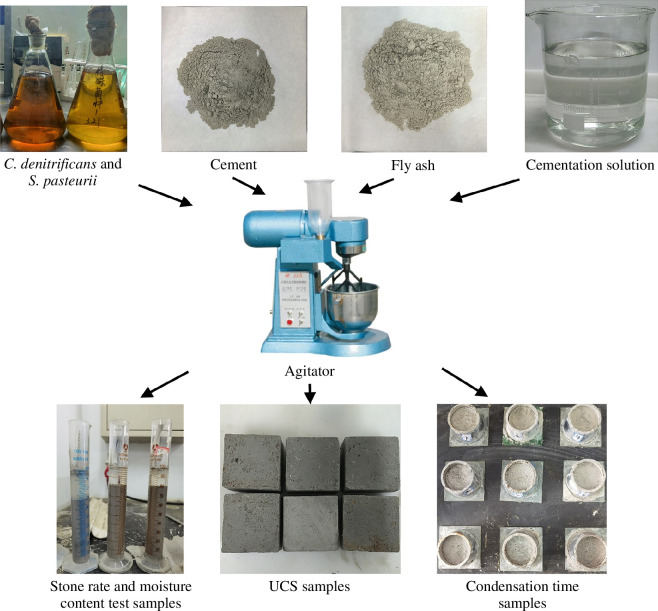
Sample preparation diagram.

After 24 h of cultivation, the *S. pasteurii* bacterial liquid was taken out and set it aside. The *C. denitrificans* bacterial liquid was taken out and centrifuged, the supernatant discarded and different volumes of fresh liquid culture medium (formulation shown in table 1) were added to obtain bacterial liquids of different concentrations. The two bacterial liquids were mixed with the cementing solution in equal volumes of 1 : 1 : 1 to form the bonding solution. Fly ash and cement were added to the cement paste mixer in proportion, and the bonding solution was added in batches at a bonding solution-to-solid mass ratio of 3 : 7. Deionized water was added to reach the predetermined water amount, and the mixer was set to a speed of 285 ± 10 r.p.m. and mixed thoroughly. Finally, the mixed slurry was poured into 50 × 50 × 50 mm triplex moulds (thinly coated with a layer of vaseline for easy demoulding) for subsequent UCS testing, with the remaining slurry used for various physical property tests (as shown in figure 2). To ensure an anoxic environment for curing the samples, the moulds were sealed with plastic wrap and placed in sealed bags.

## 3. Experimental methods

In this study, a three-factor three-level orthogonal experiment was conducted to investigate the effects of different factors on the physical and mechanical properties of the grout material. The three factors were the water–solid ratio, the solid ratio and the denitrifying bacteria concentration. Each factor was set at three levels. The water–solid ratio (*ψ*) is the ratio of the total mass of liquid to the total mass of solid in the slurry, calculated according to equation (3.1). The solid ratio (*φ*) is the ratio of the mass of cement to the mass of fly ash in the slurry, calculated according to equation (3.2).


(3.1) $$\psi =\frac{W_{\text{w}}}{W_{\text{e}}+W_{\text{c}}}.$$

(3.2) $$\varphi =\frac{W_{\text{c}}}{W_{\text{e}}}.$$

In the formulae, *W*_w_ is the mass of the liquid in the slurry, including the bacterial liquid, cementing solution (hereafter collectively referred to as the bonding solution) and deionized water. *W*_e_ is the mass of the fly ash in the slurry and *W*_c_ is the mass of the cement used to prepare the slurry. All three are measured in grams (g).

According to the ‘Technical Specifications for the Design and Construction of Goaf Highway Filling in China’ (JTGTD31-03-2011), the water–solid ratio of the grouting slurry should be between 1 : 1.0 and 1 : 1.3, and the solid ratio should be between 15% and 30%. To test the potential of microbial slurry binders, water–solid ratios of 1 : 0.8, 1 : 1.0 and 1 : 1.2 and solid ratios of 5%, 10% and 15% were selected. Considering the cost of culture, the concentration of denitrifying bacteria characterized by the absorbance of the bacterial solution at 600 nm was taken to be 0.6, 0.8 and 1.0 (OD_600_ values here and in the following text). The orthogonal test programme is shown in table 4.

**Table 4.** Orthogonal experimental design table.

specimen	water–solid ratio	solid ratio	OD_600_ values of *C. denitrificans*
1	1 : 0.8	5(95)	0.6
2	1 : 0.8	10(90)	0.8
3	1 : 0.8	15(85)	1.0
4	1 : 1.0	5(95)	0.8
5	1 : 1.0	10(90)	1.0
6	1 : 1.0	15(85)	0.6
7	1 : 1.2	5(95)	1.0
8	1 : 1.2	10(90)	0.6
9	1 : 1.2	15(85)	0.8

In this study, instruments such as a digital liquid densimeter, ZNN-D6S six-speed rotational viscometer, Vicat apparatus and electronic universal testing machine were used to measure the physical and mechanical properties of the slurry, including density, plastic viscosity, precipitation rate, stone rate, setting time and UCS. The experiments were conducted in accordance with standards such as ‘JJG999-2018 Calibration Regulation for Gravimetric Digital Liquid Densimeter in China’^2^, ‘GB/T1346-2011 Test Methods for Water Requirement of Normal Consistency, Setting Time and Soundness of the Cement in China’^3^ and ‘JGJ/T233−2011 Technical Specification for Mix Proportion Design of Cement Stabilized Soil in China’^4^.

## 4. Results

### 4.1. Experimental results

The experimental results of the physical and mechanical properties of the slurry are shown in table 5, including six physical indicators: density, plastic viscosity, initial setting time, final setting time, stone rate and precipitation rate, as well as two mechanical indicators: 7 and 28 days UCS (hereafter referred to as 7 and 28 d). To investigate the primary and secondary order and significance of the influence of denitrifying bacteria concentration, water–solid ratio and solid ratio on the above indicators, range analysis and variance analysis were further conducted on each indicator.

**Table 5.** Experimental results.

specimen	density (g cm^−3^)	plastic viscosity (mPa·s)	initial setting time (h)	final setting time (h)	stone rate (%)	precipitation rate (%)	7 d UCS (MPa)	28 d UCS (MPa)
1	1.24	6.50	170.82	205.13	58.62	37.24	0.023	0.081
2	1.28	6.80	158.12	177.56	61.25	34.88	0.60	0.73
3	1.30	7.00	113.98	133.13	73.22	24.10	0.88	1.50
4	1.34	8.90	140.33	183.00	83.59	14.77	0.15	0.23
5	1.38	9.20	131.76	163.78	83.67	14.70	0.58	0.69
6	1.40	9.50	96.19	126.91	84.00	14.40	0.76	1.44
7	1.41	13.80	104.52	125.84	90.53	8.52	0.19	0.20
8	1.43	14.00	97.46	127.31	91.79	7.39	0.64	0.76
9	1.46	14.30	67.20	96.00	90.63	8.43	1.13	2.02

### 4.2. Range analysis of physical and mechanical properties

**Figure 3.**
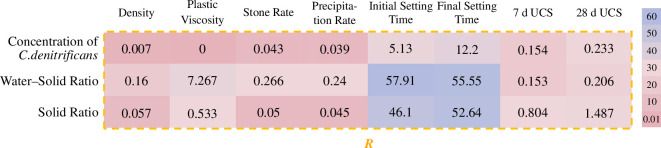
Range analysis of orthogonal experimental results.

In this study, correlation analysis was first carried out using range analysis to determine the order of precedence of the effects of the three factors, denitrifying bacteria concentration, water–solids ratio and solid ratio, on each property of the slurry, with *R* being the range derived from the experimental results of the physico-mechanical properties, and the results of the analysis are shown in figure 3.

From figure 3, it can be observed that the primary and secondary factors influencing each physical property are, in order, the water–solid ratio, solid ratio and denitrifying bacteria concentration. The water–solid ratio has the greatest impact on all physical properties, as the physical properties of the liquid phase and solid phase (fly ash and cement) differ significantly, while the differences between the physical properties of fly ash and cement are relatively small. Consequently, the impact of the solid ratio is consistently smaller than that of the water–solid ratio, and the influence of the denitrifying bacteria concentration is minimal. It is noteworthy that although the range of denitrifying bacteria concentration is 5.13 for initial setting time, it increases to 12.20 for final setting time, which is closer to the ranges of water–solid ratio and solid-to-liquid ratio. This indicates that when the slurry is freshly prepared, the MICP reaction has not yet fully proceeded. As the reaction progresses, the production of calcium carbonate gradually increases, thereby exerting a certain effect on the physical properties of the slurry. And under the condition of water–solid ratio of 1 : 1.2 and solid ratio of 15%, the initial and final setting times were 67.2 and 96 h, respectively. Compared with the setting time derived by Cao *et al*. at similar water–solid ratio and solid ratio, the initial and final setting times of the slurry treated by the MICP method were extended by nearly 70% and 110% [26], indicating that the MICP method can increase the slurry mobility, providing more time for grouting construction in goafs.

The primary and secondary factors affecting the early and late UCS of the grouted stone bodies are, in order, the solid ratio, the denitrifying bacteria concentration and the water–solid ratio. For early strength, the range of the denitrifying bacteria concentration (0.154) is very close to the range of the water–solid ratio (0.153), both of which are much smaller than the range of the solid ratio (0.804), indicating that the solid ratio has a significantly greater impact than the denitrifying bacteria concentration and the water–solid ratio. As the cultivation time increases, the range of the denitrifying bacteria concentration at 28 days is 0.233, which is greater than the range of the water–solid ratio (0.206). This indicates that during the curing process, the denitrifying bacteria continuously produce calcium carbonate and bind the solid particles in the slurry, increasing the impact of the denitrifying bacteria concentration at 28 days.

### 4.3. Physical–mechanical property variance analysis

**Figure 4.**
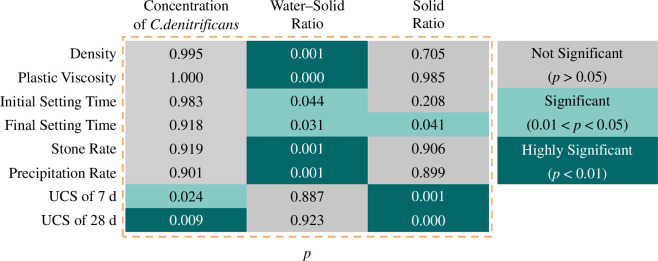
Significance graph.

Further analysis of the significance of the factors on the physical–mechanical properties of the slurry is shown in figure 4. The water–solid ratio has a highly significant correlation with slurry density, plastic viscosity, stone rate and precipitation rate and is significantly correlated with initial and final setting times. This indicates that the water–solid ratio has the highest correlation with the physical properties of the slurry, and changing the water–solid ratio will affect the initial and final setting times. This is because the amount of water significantly affects the duration of the hydration reaction of cement and fly ash. In contrast, the solid ratio is only significantly correlated with the final setting time and not significantly correlated with the other indicators. This suggests that the impact of the cement and fly ash ratio on the fluidity of the slurry increases over time, manifesting as an insignificant impact on the initial setting time but a significant impact on the final setting time. The denitrifying bacteria concentration is not significantly correlated with any of the physical properties, once again indicating that the MICP reaction has almost no impact on the physical properties of the slurry in the initial stages, consistent with the results of §4.2.

The water–solid ratio is not significantly correlated with the UCS at 7 or 28 days, consistent with the results of §4.2. The solid ratio is significantly correlated with the UCS at 7 and 28 days because an increase in the cement content relative to the fly ash content intensifies the activation reaction of the cement, interacts with the active components in the fly ash, increases the amount of calcium carbonate precipitation, and consequently increases the degree of bonding between solid particles in the grouted stone body, densifying it and increasing the compressive strength accordingly.

The denitrifying bacteria concentration is significantly correlated with the UCS at 7 days and highly significantly correlated with the UCS at 28 days. With the increase in curing time, there is a trend of increasing correlation between the denitrifying bacteria concentration and the mechanical properties. The *p*-value for the UCS at 7 days is 0.024, which decreases to 0.009 at 28 days.

### 4.4. Analysis of changes in physical properties of slurry materials

The mean values of density, plastic viscosity, setting time, stone rate and precipitation rate were analysed separately at different levels of each factor to determine their specific variations among different levels.

#### 4.4.1. Density

**Figure 5.**
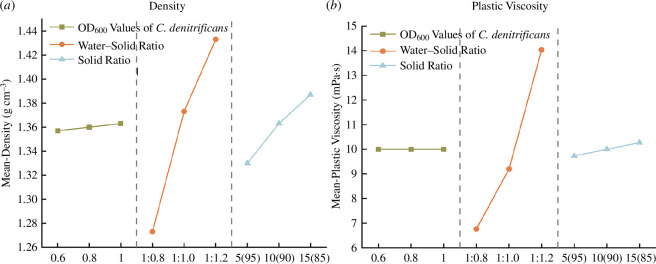
Mean values of density and plastic viscosity at different levels of each factor.

The density of different grout mixtures, as derived from the orthogonal test results, is shown in figure 5*a*. Among the factors influencing grout density, there is a positive correlation between denitrifying bacteria concentration and solid ratio with grout density, while there is a negative correlation between water–solid ratio and grout density. Within the range of factor variations set in this study, the water–solid ratio has the greatest impact on grout density while the impact of other factors is relatively weak.

#### 4.4.2. Plastic viscosity

Figure 5*b* illustrates the impact of various factors on the plastic viscosity of the slurry: the water–solid ratio is inversely correlated with the slurry’s plastic viscosity, while the solid ratio is positively correlated with it. However, changes in the concentration of denitrifying bacteria do not affect the plastic viscosity. As the water–solid ratio increases, the content of free water in the slurry increases, reducing the probability of collision between solid particles and decreasing the network-like flocculation structure formed by the mutual attraction between particles, thereby reducing the plastic viscosity of the slurry [27]. Since fly ash particles are spherical and smaller in volume than cement particles [28], a decrease in the solid ratio results in a relatively higher proportion of fly ash. Fly ash particles can be embedded between cement particles, filling some voids and improving the lubricating effect between particles. Consequently, the plastic viscosity of the slurry decreases.

#### 4.4.3. Precipitation rate and stone rate

**Figure 6.**
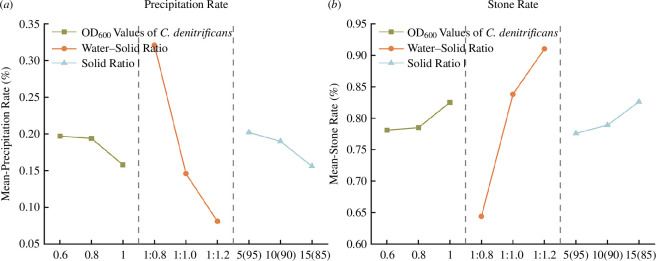
Mean values of precipitation rate and stone rate at different levels of each factor.

The precipitation rate and stone rate of slurries with different ratios are shown in figure 6. The water–solid ratio is positively correlated with the precipitation rate of the slurry, while the denitrifying bacteria concentration and the solid ratio are negatively correlated with the precipitation rate. The opposite is true for the stone rate. The water ratio is the main factor affecting the precipitation rate and stone rate, while the impact of the solid ratio and the denitrifying bacteria concentration is relatively lower.

As the water–solid ratio increases, the amount of solid phase in the slurry per unit volume decreases, leading to an increased precipitation rate and a decreased stone rate after solidification. Since the cement is more hydrophilic than fly ash, an increase in the solid ratio results in a higher proportion of cement, enhancing the hydrophilicity of the solid materials in the slurry, thereby reducing the precipitation rate and increasing the stone rate. The denitrifying bacteria concentration and the solid ratio have nearly the same level of influence on the precipitation rate and stone rate. This is because microorganisms continuously induce calcium carbonate precipitation during the resting period of the slurry, increasing the solid phase content in the slurry, ultimately leading to a decreased precipitation rate and an increased stone rate.

#### 4.4.4. Setting Time

**Figure 7.**
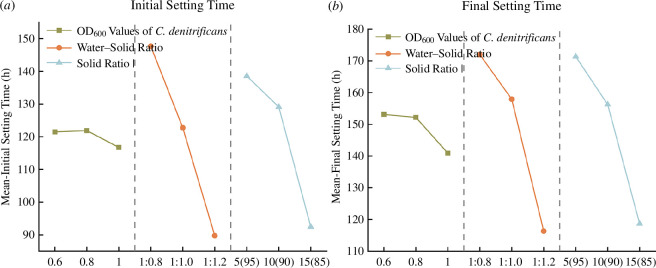
Mean values of initial setting time and final setting time at different levels of each factor.

The initial and final setting times of slurries with different ratios are shown in figure 7. The water–solid ratio is positively correlated with both the initial and final setting times of the slurry, while the denitrifying bacteria concentration and the solid ratio are negatively correlated with these setting times. Among these factors, the water–solid ratio has the most significant impact on setting times.

When the water–solid ratio increases, the distance between material particles also increases, meaning more voids need to be filled by hydration products during setting, thus prolonging both initial and final setting times. Cement contains more active components that can participate in hydration reactions than fly ash, which more readily forms C-S-H [29]. Therefore, when the solid ratio increases and the proportion of cement is higher, the slurry sets more quickly. Increasing the denitrifying bacteria concentration shortens the setting time of the slurry, as the denitrification process of denitrifying bacteria and the urea hydrolysis process of *S. pasteurii* produce calcium carbonate, which accelerates the filling of voids between particles. In an oxygen-deficient environment, denitrification predominates, resulting in shorter initial and final setting times. For sample 9, with a water–solid ratio of 1 : 1.2 and a solid ratio of 15%, the initial and final setting times are the shortest, at 67.20 and 96 h, respectively.

### 4.5. Analysis of the trends in the mechanical properties of grouting materials

**Figure 8.**
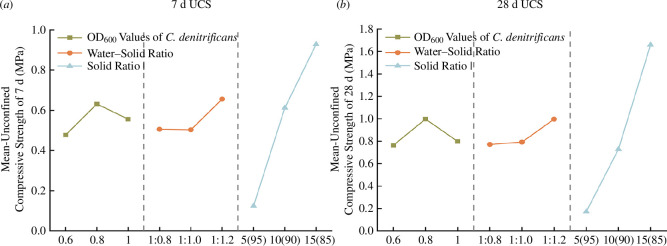
Mean values of early intensity and late intensity at different levels of each factor.

From figure 8, it can be observed that the 7 and 28 days UCS is negatively correlated with the water–solid ratio and positively correlated with the solid ratio. When the denitrifying bacteria concentration increases, the 7 and 28 day UCS initially increases and then decreases. At a denitrifying bacteria OD_600_ of 0.8, both the early and later strength averages reach their highest values of 0.63 and 0.99 MPa, respectively. When the concentration of denitrifying bacteria is too high, denitrifying bacteria continue to induce calcium carbonate precipitation in an anaerobic environment, the concentration of binder decreases too fast and the stone body gradually hardens, and the shortage of water resources and nutrients required for the subsequent MICP process leads to a decrease in the effect of the binder on the cementation of stone body particles [30], which is ultimately manifested in the optimal value of OD_600_ of denitrifying bacteria of 0.8 instead of 1.0.

## 5. Discussion

### 5.1. Comparison of the physical properties of grouting materials with other studies

**Figure 9.**
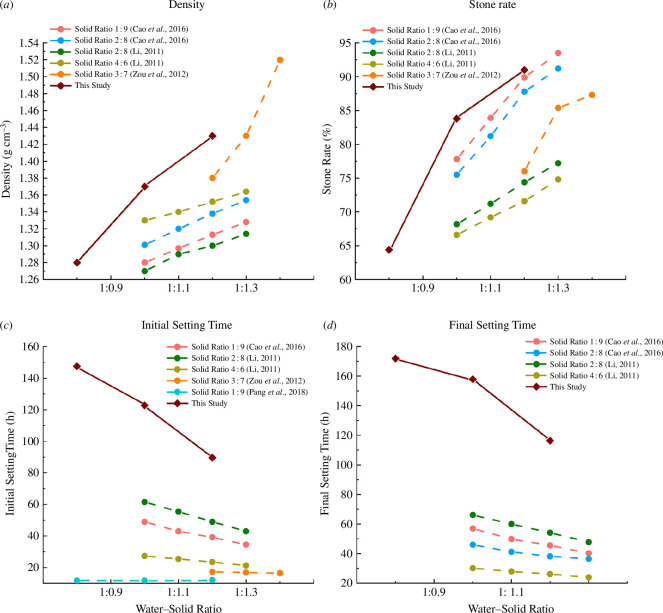
Comparison of slurry physical properties with other studies [26,31–33].

In this study, the mean values obtained for the stone rate, density and setting time obtained at different water–solid ratios were compared with those obtained in previous studies, and the results are shown in figure 9 [26,31–33]. The addition of *C. denitrificans* and *S. pasteurii* increased the density and stone rate of slurry, and significantly prolonged the setting time of slurry. The increase in density was due to the addition of nutrients required by the bacteria to the cementing solution. The dual-bacteria MICP continuously induced calcium carbonate precipitation during the sealing and maintenance process, increasing the rate of stone formation, which is in accordance with the results obtained in figure 9*b*. With the same water–solid ratio, the rate of stone formation becomes larger with the increase of fly ash incorporation. It is worth mentioning that the initial and final setting times obtained in this study were significantly prolonged compared with other studies, which further indicates that the dual-bacteria MICP method can significantly prolong the setting time of the slurry, enhance the fluidity and injectability of the slurry and provide more time for the grouting construction of the coal mine goafs.

### 5.2. Comparison of the mechanical properties of grouting materials with other studies

**Figure 10.**
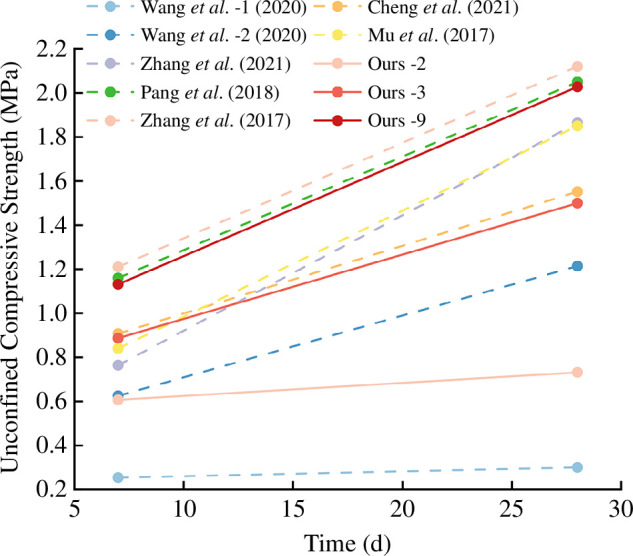
The comparison of 7 and 28 day compressive strength results of grouted stone bodies between this study and other relevant studies [32,34–38].

This study selected seven representative sets of experimental data from six related articles [32,34–38] (with two sets of data from Wang *et al*. [36] used as benchmarks) and compared them with the 7 and 28 day UCS of the second, third and ninth groups mentioned above. As shown in figure 10, the grouted stone body ratios from top left to bottom left, and top right to bottom right are C1F9W0.7, C2F8W0.7, C2F8W1+S2, C*3F7W0.8+S15, C1.25F8.75W0.35+CG25, C6.5F3.5W1+M25, C7.5F2.5W0.83+R30, C1F9W1.25+MICP0.8, C1.5F8.5W1.25+MICP1.0 and C1.5F8.5W0.83+MICP0.8. The numbers following C, F, W and MICP represent the relative content of cement, fly ash, water–solid ratio (converted to numerical form) and denitrifying bacteria concentration, respectively. C* represents the use of ultrafine fly ash; the numbers following S, CG, M and R represent the content (in percentage) of sodium silicate, coal gangue, marine clay and red mud, respectively.

Compared with other methods, the early compressive strength measured in this study’s second group under the condition of C1F9W1.25+MICP0.8 was 0.60 MPa, which is very close to the 0.62 MPa measured by Wang *et al*. [36] under the condition of C2F8W0.7. This represents a 144% increase compared with the 0.25 MPa measured under the condition of C1F9W0.7. The result measured in this study’s third group (0.88 MPa) is able to approach or even exceed the compressive strength observed in studies with lower water–solid ratios and higher relative cement content, such as 0.91 MPa under the condition of C6.5F3.5W1+M25, 0.84 MPa under C7.5F2.5W0.83+R30 and 0.76 MPa under C2F8W1+S2. This reflects the rapid early strength improvement of the MICP method.

Regarding the later strength, the 28 day compressive strength measured in the ninth group under the condition of C1.5F8.5W0.83+MICP0.8 reached as high as 2.03 MPa, slightly lower than the 2.12 and 2.05 MPa measured under the conditions of C1.25F8.75W0.35+CG25 and C*3F7W0.8+S15, respectively. The third group, with an increased water–solid ratio under the condition of C1.5F8.5W1.25+MICP1.0, also achieved a compressive strength of 1.50 MPa, close to the 1.55 MPa measured under the condition of C6.5F3.5W1+M25. This indicates that the MICP method can achieve high later strength under conditions of lower solid content and higher water–solid ratio while still meeting standard requirements. Thus, the MICP method can reduce cement usage, with biological binders offering significant advantages over materials like sodium silicate, coal gangue, marine clay and red mud.

Additionally, the results from the second, third and ninth groups suggest that increasing the solid content significantly improves compressive strength. It is anticipated that with further increases in the relative cement content, both the value and the rate of increase in compressive strength will continue to rise. Therefore, the application of MICP technology for grouting and filling in coal mine goafs has a dual significance, both environmentally and in terms of high performance, making it suitable for more demanding engineering conditions.

### 5.3. Analysis of the failure mechanism

**Figure 11.**
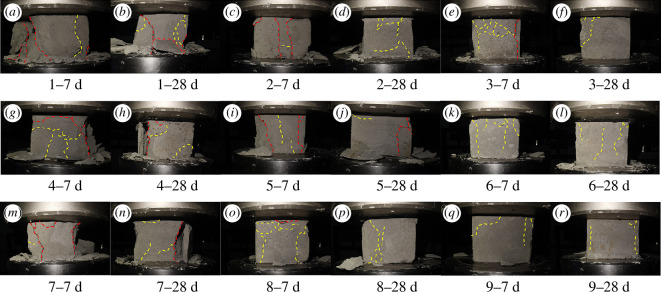
The failure modes of different samples at 7 and 28 days.

Typical failure modes of the specimens in UCS tests are shown in figure 11. The numbers before the days represent the specimen number, for example, ‘1−7 d’ represents specimen 1 at 7 days. The penetrating shear failure is marked with a red dashed line, and the semi-penetrating shear failure is marked with a yellow dashed line. Overall, most specimens exhibit obvious cracks on the surface, indicating a predominantly brittle failure mode [39]. In cases where the water–solid ratio is high and the solid ratio is low, the cracks generated during compression are wider. The failure mode of the grouted stone body is dominated by penetrating shear failure, with large areas of block detachment observed on the surface of the specimens (figure 10*a,b,g,* *h*). The instability and failure are mainly caused by the extension and penetration of the structural surface [40].

As the water–solid ratio decreases and the solid ratio increases, the failure mode transitions to semi-penetrating shear failure. This is characterized by a further reduction in block detachment of the grouted stone bodies and an increase in shear cracks on the surface of the specimens (figure 10*c,d,e,* *j*). When the water–solid ratio reaches 1 : 1.2 and the solid ratio reaches 15 (figure 10*q,* *r*), there is almost no detachment of the block bodies of the grouted stone bodies. Instead, there are small shear cracks on the surface of the specimens, with stress concentration occurring at the densest shear crack locations, indicating predominantly plastic failure.

Commonly, the failures of all specimens show a progression from the edges towards the centre, indicating that the central portion of the specimens has a higher degree of bonding and compaction than the edges. This is because the central area is less exposed to the external environment, allowing for a more thorough MICP process dominated by denitrifying bacteria, resulting in a higher degree of bonding. Additionally, new shear failures tend to develop on existing shear failures (figure 10*d,e,g,k,m,o*).

**Figure 12.**
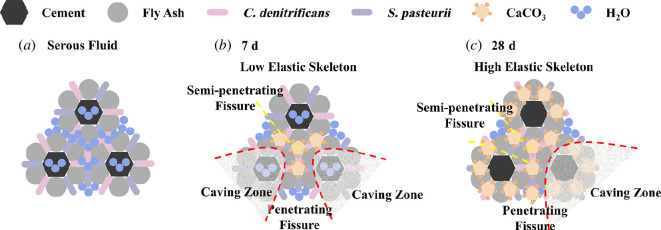
Microscopic analysis of grout failure modes.

The failure mechanism of the grout at the stages of slurry 7 and 28 days can be inferred from figure 12. It can be speculated that when the slurry is prepared, both microorganisms are evenly distributed in the slurry, mainly undergoing three processes: (i) cement hydration reaction, (ii) fly ash activation effect [41], and (iii) MICP reaction. As the curing time increases, the moisture content in the grout decreases continuously. Cement activation produces substances such as Ca(OH)_2_, and the reactive components SiO_2_ and Al_2_O_3_ in the fly ash further react with them to form hydrated compounds such as calcium silicate gel and calcium aluminate crystals. On the one hand, microorganisms continuously use themselves as nucleation sites to induce calcium carbonate precipitation, on the other hand, the substances produced by their metabolism play a regulatory role in the deposition of calcium carbonate, causing certain changes in the type and form of the deposited calcium carbonate, and continue to fill the internal voids of the stone body; the degree of solid inter-particle cementation increased, densification increased, and the strength of the skeleton and the elasticity increased accordingly [42–44].

In summary, the analysis of the effects of slurry ratio, denitrifying bacteria and curing time on the failure modes is as follows:

1. *Influence of water–solid ratio and solid ratio on failure modes*. Fly ash particles have a spherical structure and a smooth surface, so an increase in the water–solid ratio and a decrease in the solid ratio will increase the flowability of the grout, reduce viscosity and internal frictional resistance, increase the relative distance between solid particles, and weaken the bonding effect [45]. This leads to an increase in the opening angle of cracks during compression failure, making it easier to form penetrating shear failures between large particle skeletons, resulting in an increase in the number and area of collapse zones. As the water–solid ratio decreases and the solid ratio increases, the hydration reaction of the grouted stone bodies relatively intensifies, the bonding between particles becomes denser, the overall integrity improves, and the ability to resist deformation strengthens. Failure occurs in weaker bonding areas, mainly exhibiting semi-penetrating shear failure with smaller crack openings.
2. *Influence of denitrifying bacteria concentration on failure modes*. A decrease in denitrifying bacteria concentration leads to a reduction in calcium carbonate precipitation and a weakening of the bonding between solid particles. The average compressive strength of specimens reaches its maximum when the denitrifying bacteria concentration is 0.8 instead of 1.0. When the denitrifying bacteria concentration is too high, the decrease in the bonding agent concentration occurs too quickly while the hardening of the stone body is accelerated, resulting in a shortage of raw materials for the subsequent MICP process [30]. This reduces the bonding effect of the bonding agent on the particles of the grouted stone bodies, thereby affecting the failure mode of the stone bodies.
3. *Influence of curing time on failure modes*. With the increase in curing time, calcium carbonate is more easily formed in a pentagonal shape between the fly ash–cement skeleton under the alkaline conditions of the stone bodies [46]. At the same time, MICP process produces various types and properties of calcium carbonate precipitation to fill the inter-particle gap, so that the contact between the particles from the point of contact to line contact or surface contact [20]. Both MICP and the hydration reaction consume water and accelerate the hardening of the stone body. This further enhances the density and elasticity of the skeleton. Therefore, during compression failure, it mainly manifests as semi-penetrating shear failure after the strain reaches a higher value.

## 6. Conclusion

This study investigated the cementation effect of MICP technology on fly ash-based grouting materials under anaerobic or anoxic conditions using a combination of *C. denitrificans* and *S. pasteurii*. Through microbial preparation, physical-mechanical tests, comparison with other studies and analysis of failure mechanisms, the following three main conclusions were drawn:

1. The influencing factors of slurry density, plastic viscosity, stone rate, precipitation rate and setting time are in the order of water–solid ratio, solid ratio and denitrifying bacterial concentration. The denitrifying bacterial concentration is not significantly correlated with the various physical properties. However, the presence of bacteria significantly prolonged the setting time and enhanced the injectability of the slurry compared with the cement–fly ash slurry without *C. denitrificans* and *S. pasteurii*.
2. The influencing factors of early and late compressive strength of stone bodies are in the order of solid ratio, denitrifying bacterial concentration and water–solid ratio. As the cultivation time increases, the influence of denitrifying bacterial concentration on cementation effect exceeds that of water–solid ratio in the later stages. MICP and hydration reaction consumes water, accelerates the hardening of the grouted stone body, and enhances the inter-particle embedding effect through the precipitated calcium carbonate, which makes the MICP method of curing the nodular body of the pre-strength of the rapid increase in the late compressive strength of up to 2.03 MPa under the conditions of low solids ratio and high water-to-solids ratio, to meet the specification requirements. The biological bonding performance is more prominent than that of materials such as water glass, coal gangue, marine clay and red mud.
3. Increasing the water–solid ratio and decreasing the solid ratio will cause the relative distance between solid particles in the stone body to increase and the cementation effect to weaken, leading to an increase in the angle of crack opening during uniaxial compression and causing penetrating shear failure between large particle skeletons. As the water–solid ratio decreases, the solid ratio increases, and the curing time increases, cracks with small opening angles mainly occur at weakly bonded areas, resulting in semi-penetrating shear failure. The average compressive strength of samples reaches its maximum when the denitrifying bacterial concentration is 0.8. The dual-bacteria MICP mainly enhances the performance of stone bodies during curing by increasing their cohesion and elastic modulus, increasing the friction between particles, and enhancing the performance through the embedding action of calcium carbonate between the particles of the slurry.

## Data Availability

The research data supporting this paper are available from the Dryad Digital Repository [47].

## Footnotes

^1^
Shanxi Provincial Transportation Planning, Survey and Design Institute, CCCC Second Highway Survey and Design Institute Co, China University of Geosciences, China Communication Power Construction Co. Technical Specifications for Highway Design and Construction in Goafs. Industry Standards - Transport 2011:127P.;A124.

^2^
PRC State Administration of Quality Supervision and Quarantine. Calibration Regulation for Gravimetric Digital Liquid Densimeter in China. PRC State Administration of Quality Supervision and Quarantine 2018:21.

^3^
China General Research Institute of Building Materials (CGRIM), Xiamen Aceo Standard Sand Co, Zhejiang Zhongfu Construction Group Co. Test Methods for Water Requirement of Normal Consistency, Setting Time and Soundness of the Cement in China. General Administration of Quality Supervision, Inspection and Quarantine of the People’s Republic of China;Standardization Administration of China (SAC) 2011:12.

^4^
Fujian Academy of Building Research, Fujian Construction Group Corporation, Tongji University, Tianjin Academy of Building Research, Shaanxi Academy of Building Research, Zhejiang Architectural Design & Research Institute Co. Technical Specification for Mix Proportion Design of Cement Stabilized Soil in China. Industry Standards - Construction Industry 2011:60P.;B65.

## References

[1] Zhou Y , Zhang T , Duan L , Li J . 2022 Summary of research on comprehensive treatment of mine goaf in china. Saf. Environ. Eng. **29** , 220–230. (10.13578/j.cnki.issn.1671-1556.20220161)

[2] Sun Y , Bi R , Sun J , Zhang J , Taherdangkoo R , Huang J , Li G . 2022 Stability of roadway along hard roof goaf by stress relief technique in deep mines: a theoretical, numerical and field study. Geomech. Geophys. Geo-energ. Geo-resour. **8** . (10.1007/s40948-022-00356-8)

[3] Qin Y , Xu N , Zhang Z , Zhang B . 2021 Failure process of rock strata due to multi-seam coal mining: insights from physical modelling. Rock Mech. Rock Eng. **54** , 2219–2232. (10.1007/s00603-021-02415-0)

[4] Zhang JY , Wang HF , Zhang B , Li HJ , Chen QT , Li W . 2013 Exploration of mining goaf and comprehensive control technology of safety hidden dangers. Coal Sci. Technol. **41** , 76–80. (10.13199/j.cnki.cst.2013.10.028)

[5] He J , Yan HT , Song W . 2023 Discussion on the selection of ground filling materials for goaf. Shandong Coal Sci. Technol. **41** , 214–216.

[6] Dong XB , Li X , Su DL , Chen XY , Tang MX , Cheng HJ , Zhan QS , Xie XR . 2023 Research status and prospect of grouting materials and technology in geotechnical engineering. Guangzhou Arch. **51** , 149–152.

[7] Fang P , Tang Z , Zhong P , Huang J , Zeng W , Cen C . 2017 A study on leaching characteristics of heavy metals in sludge incineration slag. Chem. Ind. Eng. Prog. **36** , 2304–2310.

[8] Hemalatha T , Ramaswamy A . 2017 A review on fly ash characteristics – towards promoting high volume utilization in developing sustainable concrete. J. Clean. Prod. **147** , 546–559. (10.1016/j.jclepro.2017.01.114)

[9] Ruan S , Liu L , Zhu M , Shao C , Xie L , Hou D . 2023 Application of desulfurization gypsum as activator for modified magnesium slag-fly ash cemented paste backfill material. Sci. Total Environ. **869** , 161631. (10.1016/j.scitotenv.2023.161631) 36657671

[10] Pan X , Chu J , Yang Y , Cheng L . 2020 A new biogrouting method for fine to coarse sand. Acta Geotech. **15** , 1–16. (10.1007/s11440-019-00872-0)

[11] Tang C-S , Yin L-y , Jiang N-j , Zhu C , Zeng H , Li H , Shi B . 2020 Factors affecting the performance of microbial-induced carbonate precipitation (MICP) treated soil: a review. Environ. Earth Sci. **79** , 1–23. (10.1007/s12665-020-8840-9)

[12] van Paassen LA , Ghose R , van der Linden TJM , van der Star WRL , van Loosdrecht MCM . 2010 Quantifying biomediated ground improvement by ureolysis: large-scale biogrout experiment. J. Geotech. Geoenviron. Eng. **136** , 1721–1728. (10.1061/(ASCE)GT.1943-5606.0000382)

[13] Cheng XH , Ma Q , Yang Z , Zhang ZC , Li M . 2013 Dynamic response of liquefiable sand foundation improved by bio-grouting. Chin. J. Geotech. Eng. **35** , 1486–1495.

[14] Hassanin A , El-Nemr A , Shaaban HF , Saidani M , Shaaban IG . 2024 Coupling behavior of autogenous and autonomous self-healing techniques for durable concrete. Int. J. Civ. Eng. **22** , 925–948. (10.1007/s40999-023-00931-4)

[15] Jongvivatsakul P , Janprasit K , Nuaklong P , Pungrasmi W , Likitlersuang S . 2019 Investigation of the crack healing performance in mortar using microbially induced calcium carbonate precipitation (MICP) method. Constr. Build. Mater. **212** , 737–744. (10.1016/j.conbuildmat.2019.04.035)

[16] Rodriguez-Navarro C , Rodriguez-Gallego M , Ben Chekroun K , Gonzalez-Muñoz MT . 2003 Conservation of ornamental stone by Myxococcus xanthus-induced carbonate biomineralization. Appl. Environ. Microbiol. **69** , 2182–2193. (10.1128/AEM.69.4.2182-2193.2003) 12676699 PMC154787

[17] Kang CH , Kwon YJ , So JS . 2016 Bioremediation of heavy metals by using bacterial mixtures. Ecol. Eng. **89** , 64–69. (10.1016/j.ecoleng.2016.01.023)

[18] Ji YC . 2022 Study on mechanism of antioxidant foamed gel for preventing spontaneous combustion of coal in gob. PhD thesis, University of Science and Technology Beijing, China.

[19] Jain S , Arnepalli DN . 2019 Biochemically induced carbonate precipitation in aerobic and anaerobic environments by Sporosarcina pasteurii. Geomicrobiol. J. **36** , 443–451. (10.1080/01490451.2019.1569180)

[20] Li G , Jin C , Feng Q , Wang Q , Lu Y , Han T , Liu D . 2023 Experimental study on cemented tailings backfill based on microbially induced calcite precipitation. J. Mater. Civ. Eng. **35** , 04022476. (10.1061/(ASCE)MT.1943-5533.0004639)

[21] Hamdan N , Kavazanjian E , Rittmann BE , Karatas I . 2017 Carbonate mineral precipitation for soil improvement through microbial denitrification. Geomicrobiol. J. **34** , 139–146. (10.1080/01490451.2016.1154117)

[22] Wang Z , Su JF , Ali A , Gao ZH , Zhang RJ , Li YF , Yang WS . 2023 Microbially induced calcium precipitation driven by denitrification: performance, metabolites, and molecular mechanisms. J. Environ. Manage. **338** , 117826. (10.1016/j.jenvman.2023.117826) 37001427

[23] Harkes MP , van Paassen LA , Booster JL , Whiffin VS , van Loosdrecht MCM . 2010 Fixation and distribution of bacterial activity in sand to induce carbonate precipitation for ground reinforcement. Ecol. Eng. **36** , 112–117. (10.1016/j.ecoleng.2009.01.004)

[24] Feng QJ , Jin CY . 2023 Effect of bacterial concentration and ratio on solidification of tailings by MICP technology. Met. Mine. **563** , 333–339. (10.19614/j.cnki.jsks.202305038)

[25] Xu TC , Zhang HN , Jia CQ , W. G. H . 2023 Experimental study on disintegration properties of microbially induced calcite precipitation modified loess. Bull. Chin. Ceram. Soc. **42** , 674–681. (10.16552/j.cnki.issn1001-1625.20230207.001)

[26] Cao X , Liu X , Wu B . 2013 Cement-fly ash slurry test and its application in grouting in the hollow zone. West-China Explor. Eng. **25** , 4–7. (10.3969/j.issn.1004-5716.2013.06.002)

[27] Chen L . 2022 Influence of tailings particle grades on the rheological properties and mechanical strength of cement and steel slag-based composite filling slurries. Min. Res. Dev. **42** , 113–118. (10.13827/j.cnki.kyyk.2022.08.028)

[28] Grzeszczyk S , Lipowski G . 1999 Rheological properties of cement pastes containing fly ash. In Int. Conf. on Engineering Rheology (ICER 99), pp. 215–220. Zielona Gora, Poland.

[29] Wang J , Zhang Y , Lu L , Luo Q , Ma L , Zhang R , Li B , Li H , Liu F . 2023 Understanding the role of nano-C-S-H-polycarboxylate composites on compressive strength and frost resistance of Portland-sulfoaluminate cement blended concretes. Mat. Today. Commun. **35** , 106079. (10.1016/j.mtcomm.2023.106079)

[30] Hammad N , Elnemr A , Shaaban IG . 2023 State-of-the-art report: the self-healing capability of alkali-activated slag (AAS) concrete. Materials **16** , 4394. (10.3390/ma16124394) 37374577 PMC10302236

[31] LI H . 2011 Experiment on mixing ratio of grouting material for treatment of coal mine goaf. J. Highw. Transp. Res. Dev. **28** , 35–40. (10.3969/j.issn.1002-0268.2011.08.007)

[32] Pang JY , Yao WJ , Wang LY . 2018 Orthogonal test and regression analysis of ultrafine fly ash grouting and filling materials in the goaf. J. Yangtze River Sci. Res. Inst. **35** , 103–108. (10.11988/ckyyb.20170283)

[33] Zou Y , Zhang H , Zhang G , Cui F . 2012 Main properties test of flyash-cement grout material. Coal Min. Techn. **17** , 15–16. (10.13532/j.cnki.cn11-3677/td.2012.04.001)

[34] Cheng Q , Guo Y , Dong C , Xu J , Lai W , Du B . 2021 Mechanical properties of clay based cemented paste backfill for coal recovery from deep mines. Energies **14** , 5764. (10.3390/en14185764)

[35] Mu MG , Gao XZ , Guo TM , Hu XP . 2018 Experimental study of goaf filling materials based on red mud. In 5th Annual Int. Conf. on Material Science and Environmental Engineering (MSEE), vol. **301** , p. 012055, Xiamen, China. (10.1088/1757-899X/301/1/012055)

[36] Wang XD . 2020 Experimental study on the performance of goaf filling materials with high content of fly ash. In 3rd Int. Workshop on Environment and Geoscience (IWEG), vol. **569** , p. 012014, Electr Network. (10.1088/1755-1315/569/1/012014)

[37] Zhang KC , Zhang C , Wang DQ , Li Y , Song HT . 2021 Application of high content flyash grouting material in goaf treatment. Nonf. Met. **73** , 39–46. (10.3969/j.issn.1671-4172.2021.02008)

[38] Zhang XG , Lin J , Liu JX , Li F , Pang ZZ . 2017 Investigation of hydraulic-mechanical properties of paste backfill containing coal gangue-fly ash and its application in an underground coal mine. Energies **10** , 1309. (10.3390/en10091309)

[39] Qin Y , Tian H , Xu NX , Chen Y . 2020 Physical and mechanical properties of granite after high-temperature treatment. Rock Mech. Rock Eng. **53** , 305–322. (10.1007/s00603-019-01919-0)

[40] Qin Y , Xu N , Han J , Zhou W . 2023 Experimental study on the effects of geometric parameters of filled fractures on the mechanical properties and crack propagation mechanisms of rock masses. Rock Mech. Rock Eng. **56** , 2697–2716. (10.1007/s00603-022-03198-8)

[41] Zhu B , Yang Q , Wu X . 2002 Study of volcanic ash reactivity of I grade fly ash. China Concr. Cement Products **123** , 3–6. (10.19761/j.1000-4637.2002.01.001)

[42] Hammad N , El-Nemr A , Shaaban IG . 2024 The efficiency of calcium oxide on microbial self-healing activity in alkali-activated slag (AAS). Appl. Sci. **14** , 5299. (10.3390/app14125299)

[43] Shaaban S , Hammad N , Elnemr A , Shaaban IG . Efficiency of bacteria-based self-healing mechanism in concrete. Mater. Sci. Forum **1089** , 135–143. (10.4028/p-tc6w54)

[44] Zhang J , Xu S , Feng T , Zhao L , Li Z . 2019 Effect of mineralized bacteria type on concrete crack self-healing capacity. J. Tsinghua Univ. **59** , 607–613. (10.32604/cmc.2019.04589)

[45] Hu H , Gan B , Deng C , Xie Z , Lu Y , Cai Y . 2024 Experimental study on the effect of water-cement ratios on the diffusion behavior of sand soil grouting. Bull. Eng. Geol. Environ. **83** , 80. (10.1007/s10064-024-03580-6)

[46] Zhang HN . 2019 Experimental study on microbial induced calcium carbonate precipitation technology for sand solidification. Thesis, China University of Geosciences, Beijing, China..

[47] He H *et al* . 2024 Study on the improvement of grouting stone properties in coal mine goafs using combined denitrifying bacteria. Dryad Digital Repository. (10.5061/dryad.0000000bq) PMC11421893 39323547

